# Inhibition of the intracellular domain of Notch1 results in vascular endothelial cell dysfunction in sepsis

**DOI:** 10.3389/fimmu.2023.1134556

**Published:** 2023-05-02

**Authors:** Tingyan Liu, Caiyan Zhang, Jiayun Ying, Yaodong Wang, Gangfeng Yan, Yufeng Zhou, Guoping Lu

**Affiliations:** ^1^ Department of Critical Care Medicine, Children’s Hospital of Fudan University, Shanghai, China; ^2^ Institute of Pediatrics, Children’s Hospital of Fudan University, National Children’s Medical Center, and the Shanghai Key Laboratory of Medical Epigenetics, International Co-laboratory of Medical Epigenetics and Metabolism, Ministry of Science and Technology, Institutes of Biomedical Sciences, Fudan University, Shanghai, China; ^3^ National Health Commission (NHC) Key Laboratory of Neonatal Diseases, Fudan University, Shanghai, China

**Keywords:** NICD, vascular endothelial dysfunction, melatonin, LPS (lipopolysaccharide), sepsis

## Abstract

**Background:**

Notch signaling is critical for regulating the function of vascular endothelial cells (ECs). However, the effect of the intracellular domain of Notch1 (NICD) on EC injury in sepsis remains unclear.

**Methods:**

We established a cell model of vascular endothelial dysfunction and induced sepsis in a mouse model *via* lipopolysaccharide (LPS) injection and cecal ligation and puncture (CLP). Endothelial barrier function and expression of endothelial-related proteins were determined using CCK-8, permeability, flow cytometry, immunoblot, and immunoprecipitation assays. The effect of NICD inhibition or activation on endothelial barrier function was evaluated *in vitro*. Melatonin was used for NICD activation in sepsis mice. The survival rate, Evans blue dye of organs, vessel relaxation assay, immunohistochemistry, ELISA, immunoblot were used to explore the specific role of melatonin for sepsis induced vascular dysfunction *in vivo*.

**Results:**

We found that LPS, interleukin 6, and serum collected from septic children could inhibit the expression of NICD and its downstream regulator Hes1, which impaired endothelial barrier function and led to EC apoptosis through the AKT pathway. Mechanistically, LPS decreased the stability of NICD by inhibiting the expression of a deubiquitylating enzyme, ubiquitin-specific proteases 8 (USP8). Melatonin, however, upregulated USP8 expression, thus maintaining the stability of NICD and Notch signaling, which ultimately reduced EC injury in our sepsis model and elevated the survival rate of septic mice.

**Conclusions:**

We found a previously uncharacterized role of Notch1 in mediating vascular permeability during sepsis, and we showed that inhibition of NICD resulted in vascular EC dysfunction in sepsis, which was reversed by melatonin. Thus, the Notch1 signaling pathway is a potential target for the treatment of sepsis.

## 1 Introduction

Sepsis is a life-threatening disease contributing to global health loss (1). Sepsis-induced vascular dysfunction is pivotal to the pathobiology of sepsis and leads to septic shock and even multiple organ failure (2). Children suffering from septic shock exhibit higher mortality (3). Therefore, exploring the underlying molecular mechanisms of sepsis-induced vascular dysfunction could help to improve clinical outcomes in patients with sepsis. Over the past few years, treatments for septic shock have focused on early fluid resuscitation, application of vasoconstrictive drugs, removal of the inflammatory factor waterfall released in large quantities, immune modulation with glucocorticoids, and antimicrobial and endotoxin neutralization (4). However, these treatments often fail to reverse disease progression.

Vascular endothelial cells (ECs) are the primary cells that regulate vascular function (5, 6). As an important part of the blood vessel wall, ECs are the first cells to sense the damage induced by sepsis. For example, lipopolysaccharide (LPS) produced by pathogens and cytokines can directly cause EC injury and disrupt endothelial cytoskeletal proteins as well as intercellular junctions and adhesions, resulting in increased vascular EC permeability and the diversion of fluids into extravascular tissues, leading to edema. Upregulation of endothelial nitric oxide synthase in vascular ECs generates large amounts of nitric oxide, causing vasodilation and increased expression of inducible nitric oxide synthase. Sustained elevation of nitric oxide levels in the late phase of shock affects the function of vascular smooth muscle. Damaged vascular smooth muscle cells can further lead to impaired vascular responsiveness, which makes fluid resuscitation and vasopressor therapy ineffective (7). Therefore, strategies that ameliorate or even prevent endothelial dysfunction are essential for treating septic shock.

Notch1 signaling pathway is a conserved pathway that regulates vascular EC function (8). When the Notch1 receptor is bound by one of its ligands (such as DLL4, Jagged1), the Notch1 protein is sheared by specific enzymes into extracellular and intracellular segments. The intracellular segment region (Notch intracellular domain, NICD) is transported into the nucleus where it forms a complex with DNA-binding proteins, thereby derepressing target genes, especially Hes1, and allowing them to be transcriptionally activated to fulfil their biological roles. Given that the formation of NICD is the most critical event in the activation of the Notch1 signaling pathway, the cellular level of NICD reflects the activity of Notch1 signaling (9). Previous studies have revealed that activation of Notch1 directly regulates vascular barrier function in an engineered organotypic model (10). The reduction of endothelial Notch1 was identified as a predisposing factor for the onset of vascular inflammation and initiation of atherosclerosis (11). Furthermore, loss of Notch1 in adult endothelium increases hypercholesterolemia-induced atherosclerosis in the descending aorta (12). These findings indicate that Notch1 expression is downregulated in vascular cells in response to chronic inflammation during atherosclerosis. However, the significance of Notch1 signaling, especially NICD, in sepsis-induced vascular dysfunction is unknown.

Here, we report a previously uncharacterized role of Notch1 in mediating vascular permeability during sepsis. We found that levels of NICD and its downstream regulator Hes1 in ECs were reduced during sepsis, which further impaired vascular permeability *via* the Akt signaling pathway. We propose that LPS inhibits expression of a deubiquitylating enzyme USP8 to decrease the level of NICD. We also observed that melatonin mitigated sepsis-induced EC injury by upregulating USP8, thus stabilizing NICD, providing scientific support for the application of melatonin to treat sepsis. In summary, our study suggests that NICD in the Notch1 signaling pathway is a promising target for the treatment of sepsis.

## 2 Materials and methods

### 2.1 Ethical approval of participants

Blood samples were collected from six normal controls and children diagnosed with sepsis shock between January 2020 and December 2020 from the pediatric intensive care unit (PICU) of the Children’s Hospital of Fudan University, China. Septic shock in children is defined as a severe infection that leads to cardiovascular dysfunction, including hypotension requiring treatment with vasoactive medication, or impaired perfusion (13). Blood collection was performed on the day of admission to the PICU, and samples were centrifuged at 400 g for 10 min to separate serum in the supernatant. Written informed consent was obtained from all participants. The study was approved by the Research Ethics Board in the Children’s Hospital of Fudan University (IRB protocol number: 2020-424). General patient information and representative laboratory data are listed in **Table 1**.

**Table 1:** Characteristics of serum from septic shock children used for cell stimulation.

Patient No.	Age (months)	Gender	Pathogen	Infection source	IL-6 (pg/mL)	LPS (EU/mL)	PCIS score	Clinical outcome
1	19	Male	/	Central nervous system	0.0135	0.0254	84	Dead
2	33	Female	Acinetobacter baumannii	Abdominal	288.9	0.129	86	Alive
3	6	Male	/	Respiratory tract	131.5	<0.01	96	Alive
4	48	Female	Adenovirus	Respiratory tract	889.5	0.0309	90	Alive
5	23	Male	Methicillin resistant staphylococcus aureus	Skin	204.7	0.0135	90	Alive
6	108	Female	Acinetobacter baumannii	Respiratory tract	3164	0.0276	90	Dead
7	29	Female	/	Abdominal	34.76	0.0392	76	Alive

IL-6, interleukin-6; PCIS, Pediatrics Critical Illness Score.

/, culture-negative

### 2.2 Antibodies and reagents

The following primary antibodies were purchased from Cell Signaling Technology (Danvers, USA): anti-cleaved Notch1 (4147s; 1:1,000), anti-Hes1 (11988s; 1:1,000), p-eNOS (9570s, 1:1,000), eNOS (32027s, 1:1,000), iNOS (13120s,1:1,000), anti-AKT (4691s; 1:1,000), anti-phospho-AKT antibody (Ser473) (4060s; 1:1,000), anti-PTEN antibody (9559s; 1:1,000), anti-Actin antibody (4970s; 1:1,000), and anti-USP8 (11832s; 1:1,000). The following primary antibodies were obtained from Abcam (Waltham, USA): anti-USP11 (ab109232; 1:1,000), anti-VE-Cad antibody (ab33168; 1:1,000), and anti-Zo-1 antibody (ab216880; 1:1,000). LPS (L4516) and interleukin 6 (IL-6) were acquired from Sigma-Aldrich Merck KGaA (Germany). Melatonin and tangeretin (TGN) were purchased from Aladdin (purity >95%; Aladdin, China).

### 2.3 Cell culture

The human-derived vascular EC line (Ea.hy 926) was provided by the Cell Bank of the Chinese Academy of Sciences Shanghai Branch (Shanghai, China). Cells were cultured in high glucose Dulbecco’s modified Eagle’s medium (DMEM) with 1% antibiotics (Hyclone, USA) and 10% fetal bovine serum (FBS) in an incubator at 37°C with 95%/5% air/CO_2_. The authenticity of cell lines was confirmed by short tandem repeat (STR) profiling. All cell lines were passaged twice a week at a ratio of 1:4. For signaling studies, cells were cultured in serum-starved medium to avoid masking stimulation signals. All cell lines were regularly checked for morphological changes and the presence of mycoplasma. Only mycoplasma-negative cell lines were used in subsequent experiments.

### 2.4 Western blot analysis

For immunoblotting, total protein was extracted from cells using RIPA lysis buffer (Beyotime, China). The amount of protein was quantified using the BCA protein assay kit (Beyotime, China), and 20 μg of total protein was subjected to SDS–PAGE (10%, Bio-Rad) and then transferred to a PVDF membrane (Merck Millipore, Germany). The membrane was blocked with 5% skim milk for 1 h and subsequently incubated with the indicated primary antibodies overnight at 4°C. Horseradish peroxidase-conjugated anti-rabbit antibody (1:5,000) was utilized as the secondary antibody. Protein bands were visualized using a chemiluminescent HRP substrate (ThermoFisher, USA) and imaged with a Molecular Imager^®^ ChemiDocTM XRS+ Imaging System (Bio-Rad).

### 2.5 Cell proliferation assay

Ea.hy 926 cells were seeded in 96-well culture plates at a density of 1 × 10^3^/well and stimulated for 24 h according to the experimental requirements. The rate of cell proliferation was determined using the CCK8 assay (Dojindo, Japan). Cells were incubated for 1 h and tested every 24 h using a microplate reader at 450 nm.

### 2.6 Permeability assay

A classic Transwell chamber assay was conducted to evaluate EC permeability using fluorescence-labeled dextran (40 kDa, Sigma-Aldrich Merck KGaA, Germany) (14). The assay was performed in a 12-well plate using individual polycarbonate membrane inserts with a 0.4 µm pore (Corning, USA). Ea.hy 926 cells were seeded at a density of 1 × 10^5^ cells/insert. The medium in both upper and bottom compartments was replaced with fresh medium 24 h after seeding, and cells were treated according to the indicated protocols. Inserts were transferred into a new plate containing 600 μL PBS, and 60 μg of fluorescence-labeled dextran (40 kDa) diluted in 300 μL PBS was then added to the upper compartment of the insert. Inserts were cultured for 1 h in the incubator, and then PBS from the lower chamber was transferred to a plate reader (BioTek, Synergy2) and measured (excitation: 480 nm; emission: 510 nm).

### 2.7 Wound healing assay

Ea.hy 926 cells seeded in 6-well cell culture plates were scraped by P10 tips and then incubated with serum-free medium. Images of wells under the same view were obtained using a Leica Microscope immediately and 24 h after scratch. The rate of cell migration, calculated as the average percent of wound closure, was determined using Image J.

### 2.8 Cell apoptosis assay

Cell apoptosis was determined using an AnnexinV-fluorescein isothiocyanate (FITC)/propidium iodide (PI) double-staining kit (BD Biosciences, USA), according to the manufacturer’s instructions. Ea.hy 926 cells were cultured in 6-well plates (1 × 10^6^ cells/well) for 24 h. After treatment with LPS and melatonin, the cells were digested with ethylenediaminetetraacetic acid (EDTA)-free trypsin, stained with AnnexinV-FITC and PI, and subjected to flow cytometry using FACSCanto™ II (BD Biosciences). FlowJo V10 software (Tree Star, USA) was used for data analysis. Cells that were negative for both Annexin V-FITC and PI were considered viable. Annexin V-FITC positive and PI negative cells were recognized as early apoptosis; Annexin V-FITC and PI positive cells were late apoptosis; and Annexin V-FITC negative and PI positive cells were necrotic cells. Early and late apoptotic cells were used to calculate the percentage of apoptotic cells.

### 2.9 Plasmid and siRNA transfection

The expression plasmid containing NICD sequence (aa 1757-2555) was purchased from Genechem (China) and transfected into Ea.hy 926 cells using Lipofectamine 3000 reagents (Invitrogen, USA). Stable clones were selected using puromycin at a concentration of 2 μg/mL for two weeks. siRNA targeting USP8 and scramble control siRNA were purchased from RiboBio (China) and transfected using Lipofectamine 3000 reagent. The siRNA sequences were: si-USP8 1# (human) (5’-CTGGAACCTTTCGTTATTA-3’), si-USP8 2# (human) (5’- TCATCTCGATGACTTTAAA-3’), and si-USP8 3# (human) (5’- CTACGATGGCAGGTGGAAA-3’). The efficiency of si-USP8 transfection was evaluated using western blot analysis.

### 2.10 Ubiquitination and immunoprecipitation (IP) assay

Ea.hy 926 cells were lysed with 1% SDS in lysis buffer for the ubiquitination assay or RIPA lysis buffer for the IP assay. The cell lysates were diluted to 1 mg/mL and mixed with the indicated primary antibody and then incubated at 4°C overnight. Protein A/G magnetic beads were added to the lysate-antibody mixture and incubated for 2 h at room temperature. Western blot analysis was performed to detect ubiquitin modification or IP proteins as described above.

### 2.11 Quantitative real-time PCR

Total RNA from aortic tissue samples of mice was extracted using Trizol (Invitrogen, USA) following the manufacturer’s protocol. A total of 1 μg RNA was reverse-transcribed into cDNA using the Primer Script RT reagent kit (Takara, Japan). *Gapdh* and *Actin* were used as endogenous standards. Triplicate PCR reactions were performed, and the relative expression levels were calculated using the 2^-△△Ct^ equation. The sequences of all primers are provided in **Supplemental Table 1.**

### 2.12 Assay for NICD stability

Ea.hy 926 cells were pretreated with 20 μM cycloheximide (CHX, Sigma-Aldrich Merck KGaA, Germany), an inhibitor of protein synthesis, and were collected at different time points (0, 2, 4, and 6 h) after indicated treatments. NICD expression was determined using Western blot analysis.

### 2.13 Animals

Male C57BL/6 mice aged 3-4 weeks were obtained from Shanghai Slake Laboratory Animal Company (China). All animals were housed at 25°C, with a 12-h light/12-h dark cycle under specific pathogen-free conditions and free to food and water. Experiments were conducted one week after the mice adapted to the new environment. The experimental protocols used in this study were approved by the Animal Studies Committee of the Children’s Hospital of Fudan University (IRB protocol number: 2019-321).

### 2.14 Animal models and melatonin administration

For LPS-induced-sepsis models, mice were injected intraperitoneally with one dose of LPS at 10 mg/kg body weight, and saline was administered to the controls. For survival analysis, LPS at 15 mg/kg body weight was used for LPS-induced sepsis model. The mouse model of polymicrobial sepsis was induced by CLP as previously described (15). Mice were fasted for 12 h before surgery and were anesthetized with tribromoethanol. The midline laparotomy was performed after shaving the skin and disinfection, and 50% of the distal end of the cecum was ligated. A single through-and-through puncture was implemented using 22-gauge needles between the end of the cecum and the ligation site, and then a small amount of feces was squeezed out through the puncture. The cecum was returned to the abdominal cavity. Abdominal musculature and skin incision were closed with sutures. The animals undergoing laparotomy and cecum exposure without ligation and puncture were used as sham-operated controls. All mice were revived with 1 mL of saline.

Once the sepsis models were established, mice in the melatonin treatment group were injected intraperitoneally for LPS-induced sepsis mice and subcutaneously for CLP mice with melatonin dissolved in saline at a dose of 30 mg/kg, which was modified based on our pilot experiments and previous literature (16). Based on the half-time of melatonin *in vivo*, repeated administration of the drug was performed. All experiments were conducted three times.

### 2.15 Immunohistochemistry

Mice were sacrificed at 24 h post-surgery/LPS administration under anesthesia followed by blood and organ collection. Fresh aorta samples were first fixed in 4% paraformaldehyde and then embedded in paraffin. For immunohistochemistry assays, tissues were sectioned at 5 μm and then incubated in citrate antigen retrieval solution (pH = 6.8) for 15 min at 95°C. The sections were then stained with NICD antibody (Abcam) at 4°C overnight. Next, the sections were incubated with corresponding HRP-conjugated secondary antibodies (Absin, China) for 1 h at room temperature and were visualized using diaminobenzidine (Absin, China).

### 2.16 Enzyme-linked immunosorbent assay (ELISA)

Mouse serum was collected for cytokines detection using a mouse TNF-α ELISA kit (JL10484, Jianglai, Shanghai, China), a mouse IL-6 ELISA kit (JL20268, Jianglai, Shanghai, China) and a mouse melatonin ELISA kit (JL10087, Jianglai, Shanghai, China) according to the manufacturer’s instructions.

### 2.17 Measurement of lactate

Lactate levels in the serum of mice were determined using a lactate assay kit (Nanjing Jiancheng Bioengineering Institute, China) according to the manufacturer’s instructions.

### 2.18 Endothelium-dependent relaxation function of vessels assay

The thoracic aorta was carefully dissected, cleaned of fat and adherent connective tissues, and then cut into 2–3 mm vessel rings and mounted on two stainless-steel stirrups in the 10 mL organ chamber of a Multi Myograph System (Danish Myo Technology (DMT), Aarhus, Denmark) containing 5 mL PSS (1000 mL contained 7.6 g NaCl; 0.35 g KCl; 0.29 g MgSO_4_; 0.16 g KH_2_PO_4_; 1.25 g NaHCO_3_; 1 g glucose; 0.01 g Na_2_-EDTA; and 1.6 mL of 1 mol/L CaCl_2_). The PSS was kept at 37°C and bubbled continuously with 95% O_2_ and 5% CO_2_. The vessel rings were stretched to a resting tension of 3.5 mN and equilibrated for 60 min with PSS buffer. After equilibration, the rings were stimulated twice with KPSS (1000 mL contained 4.37 g NaCl; 4.47 g KCl; 0.29 g MgSO_4_; 0.16 g KH_2_PO_4_; 1 g NaHCO_3_; 1 g glucose; 0.01 g Na_2_-EDTA, and 1.6 mL of 1 mol/L CaCl_2_) for 15 min. Vasodilatation was assessed by applying acetylcholine (Ach) (17). When a contractile plateau was reached after norepinephrine (10^-9^ to 10^-5^M), concentration-response dilations to cumulative Ach (10^-9^ to 10^-5^M) were performed. Doses were added every 3–5 min.

### 2.19 Evans blue assay for the permeability of vessels

0.5% Evans Blue dye (Solarbio, Beijing, China) was injected *via* a tail vein 30 min before the mice were killed. Subsequently, mice were perfused with PBS *via* the left ventricle to remove the intravascular dye. Lungs, livers, and hearts were removed and dried for 1 h. Evans blue dye was extracted in formamide by incubating tissues overnight at 55°C. Evans blue dye levels in tissue supernatant were measured by spectrophotometric analysis at 620 nm (18).

### 2.20 Statistical analysis

Statistical significance of the differences between groups was determined using the Student’s t-test or one-way ANOVA plus Tukey’s analysis using SPSS software (version 22.0) for comparisons of two groups or multiple groups, respectively. Kaplan-Meier survival analysis was utilized to compare survival rate between the sepsis group and treatment group. Data are presented as the mean ± standard error of the mean (SEM). *P* < 0.05 was considered statistically significant.

## 3 Results

### 3.1 NICD expression and activity are decreased *in vitro* models of sepsis

**Figure 1:**
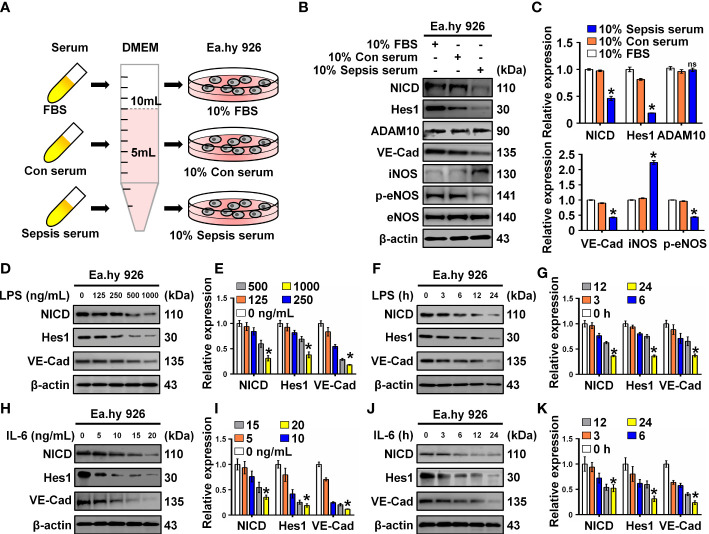
Serum from sepsis patients, LPS and IL-6 inhibit Notch signaling. **(A)** FBS, control, and sepsis serum were collected to treat Ea.hy 926 cells. **(B, C)** The expression of NICD, Hes1, ADAM10, VE-Cad, iNOS, p-eNOS, and eNOS in Ea.hy 926 cells were tested by Western blot after treating with the collected serum for 4 (h) Representative images **(B)** and three experiments replicates **(C)** were displayed. **(D, E)** The levels of NICD, Hes1 and VE-Cad were detected by Western blot in Ea.hy 926 cells stimulated with the indicating concentration of LPS for 24 (h) Representative images **(D)** and three experiments replicates **(E)** were displayed. **(F, G)** The expression of NICD, Hes1 and VE-Cad were detected by Western blot in Ea.hy 926 cells stimulated with 1000 ng/mL LPS for indicating time. Representative images **(F)** and three experiments replicates **(G)** were displayed. **(H, I)** The expression of NICD, Hes1 and VE-Cad were quantified by Western blot in Ea.hy 926 cells stimulated with the indicating concentration of IL-6 for 24 (h) Representative images **(H)** and three experiments replicates **(I)** were displayed. **(J, K)** The levels of NICD, Hes1 and VE-Cad were quantified by Western blot in Ea.hy 926 cells stimulated with 20 ng/ml IL-6 for indicating time. Representative images **(J)** and three experiments replicates **(K)** were displayed. Data are shown as the mean ± SEM. ns, no significance; *p < 0.05.

To investigate the effect of serum collected from children with sepsis on ECs, Ea.hy 926 cells were incubated with 10% septic serum in DMEM for 4 h. FBS and healthy serum at equal concentrations were used as controls (**Figure 1A**). Expression of NICD, the effector of Notch signaling, and its related proteins including Hes1, ADAM10, VE-Cad, iNOS, p-eNOS, and eNOS were detected using Western blot analysis. Compared to the control groups, cells cultured with septic serum displayed a notable decrease in the expression of NICD, Hes1, VE-Cad, and p-eNOS, whereas the expression of ADAM10 was not significantly altered. Furthermore, there was a substantial increase in the expression of iNOS in the cells treated with septic serum (**Figures 1B, C**). These results indicate that septic serum can downregulate the expression of NICD and its downstream molecules.

LPS and inflammatory cytokines, such as IL-6, in septic serum may play an essential role in inducing EC dysfunction. LPS and IL-6 have been utilized to induce *in vitro* models of sepsis in cultured ECs (19, 20). Therefore, we investigated whether LPS and IL-6 could alter the expression of NICD, Hes1, and VE-Cad. We found that NICD, Hes1, and VE-Cad expression in Ea.hy 926 cells were reduced by LPS (**Figures 1D–G**) or IL-6 (**Figures 1H–K**) in a concentration- and time-dependent manner. These results suggest that septic serum, LPS, and IL-6 can inhibit NICD and its downstream signaling molecules.

### 3.2 Inhibition of NICD impairs the endothelial barrier and leads to EC injury

**Figure 2:**
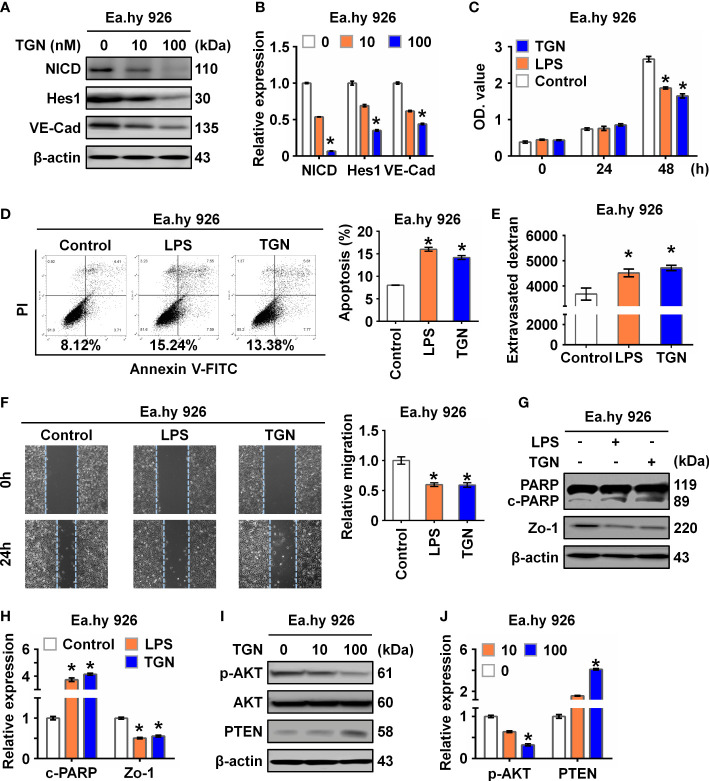
Inhibition of NICD impairs endothelial function. **(A, B)** The expression of NICD, Hes1, and VE-Cad in Ea.hy 926 cells were tested by Western blot after treating with different concentrations of TGN for 24 (h) Representative images **(A)** and three experiments replicates **(B)** were displayed. **(C)** CCK8 assay was used to evaluate the proliferation of Ea.hy 926 cells treated with LPS and TGN for 24 (h) **(D)** The apoptosis of Ea.hy 926 cells regulated by LPS or TGN was tested with Annexin V-FITC/PI staining by flow cytometry. **(E)** The endothelial permeability was determined by the transwell assay after being treated with LPS and TGN for 24 (h) Quantitative analysis was performed by measuring FD40 fluorescence intensity relative to the control. **(F)** The migration of Ea.hy 926 cells was tested by wounding healing assay after being treated with LPS and TGN for 24 (h) The percentage of wound closure was determined at 0 h and 24 (h) Representative microscopic cell images (F-left) immediately after wound creation and 24 h later and statistical analysis of migration **(F)**-right) were shown. **(G, H)** The expression of cleaved PARP and Zo-1 were tested by western blot in LPS-induced Ea.hy 926 cells with or without TGN. Representative images **(G)** and three experiments replicates **(H)** were displayed. **(I, J)** The protein levels of PTEN and p-AKT were tested in Ea.hy 926 cells treated with indicated concentration of TGN. The data were obtained from three independent experiments. Data are shown as the mean ± SEM. *p < 0.05.

To explore the role of NICD in endothelial barrier function, Ea.hy 926 cells were treated with TGN, a flavonoid that can inhibit the Notch signaling pathway (21). Consistent with a previous study (22), we found that TGN inhibited protein expression of NICD, Hes1, and VE-Cad in a concentration-dependent manner (**Figures 2A, B**), which was similar to the results of Ea.hy 926 cells treated with LPS or IL-6. Next, we analyzed the impact of LPS and TGN on endothelial barrier function and found that both TGN and LPS impaired cell proliferation (**Figure 2C**), promoted cell apoptosis (**Figure 2D**), increased cell permeability (**Figure 2E**) and inhibited cell migration (**Figure 2F**), increased the levels of cleaved PARP (an apoptosis indicator protein) and decreased Zo-1 expression (cell junction protein) in Ea.hy 926 cells (**Figures 2G, H**). These results demonstrate that NICD and Notch signaling are critical in regulating EC functions.

Since Akt signaling is known for maintaining endothelial barrier function (23), we further investigated if the Akt signaling pathway was involved in Notch signaling in our sepsis model. We found that p-AKT was decreased while PTEN, a negative regulator of p-AKT, was increased in cells treated with TGN, LPS or IL-6 (**Figures 2I, J**; **Supplementary Figures 1 A, B**), suggesting that the damage to EC function resulting from sepsis was likely mediated by the AKT pathway.

### 3.3 Overexpression of NICD alleviates LPS-induced endothelial dysfunction

**Figure 3:**
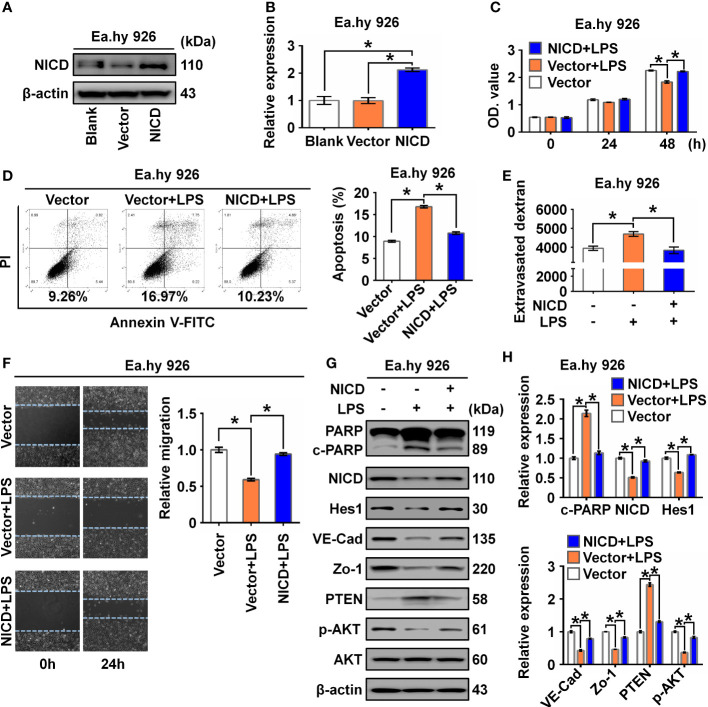
Overexpression of NICD reversed LPS-caused endothelial dysfunction. **(A, B)** The expression of NICD in Ea.hy 926 cells was tested by Western blot after transfected with blank plasmids, vector or NICD plasmids. Representative images **(A)** and three experiments replicates **(B)** were displayed. **(C)** CCK8 assay was used to evaluate the proliferation of Ea.hy 926 cells treated with the indicated conditions. **(D)** The apoptosis of Ea.hy 926 cells treated with indicated conditions was tested with Annexin V-FITC/PI staining by flow cytometry. Representative images **(D)**-left) and statistical analysis of apoptosis **(D)**-right) for Ea.hy 926 cells transfected by blank plasmids, vector or NICD plasmids after LPS treatment were shown. **(E)** The endothelial permeability was determined by the transwell assay after being treated with the indicated conditions. **(F)** The migration of Ea.hy 926 cells was tested by wounding healing assay. Representative images **(F)**-left) and statistical analysis of migration **(F)**-right) for Ea.hy 926 cells transfected by blank plasmids, vector or NICD plasmids after LPS treatment were shown. **(G, H)** The protein levels of cleaved PARP, NICD, Hes1, VE-Cad, Zo-1, PTEN, and p-AKT was tested in LPS-treated Ea.hy 926 cells with or without NICD-transfected. Representative images **(G)** and three experiments replicates **(H)** were displayed. The data were obtained from three independent experiments. Data are shown as the mean ± SEM. *p < 0.05.

To determine whether NICD overexpression could reverse or alleviate LPS-induced endothelial dysfunction, we overexpressed NICD in the Ea.hy 926 cells and confirmed the overexpression using Western blot analysis (**Figures 3A, B**). We found that NICD overexpression reversed the reduced cell viability and elevated cell apoptosis induced by LPS treatment (**Figures 3C, D**). Furthermore, the increased intercellular permeability and impaired cell migration caused by LPS were alleviated following NICD overexpression (**Figures 3E, F**). Subsequently, expression of NICD downstream genes including Hes1, VE-Cad, and Zo-1, and Akt signaling pathway molecules, such as p-AKT and PTEN, were restored following NICD overexpression (**Figures 3G, H**). These data demonstrate that NICD overexpression can alleviate EC dysfunction induced by LPS.

### 3.4 Melatonin alleviates LPS-induced endothelial dysfunction by upregulating NICD and activating AKT signaling

**Figure 4:**
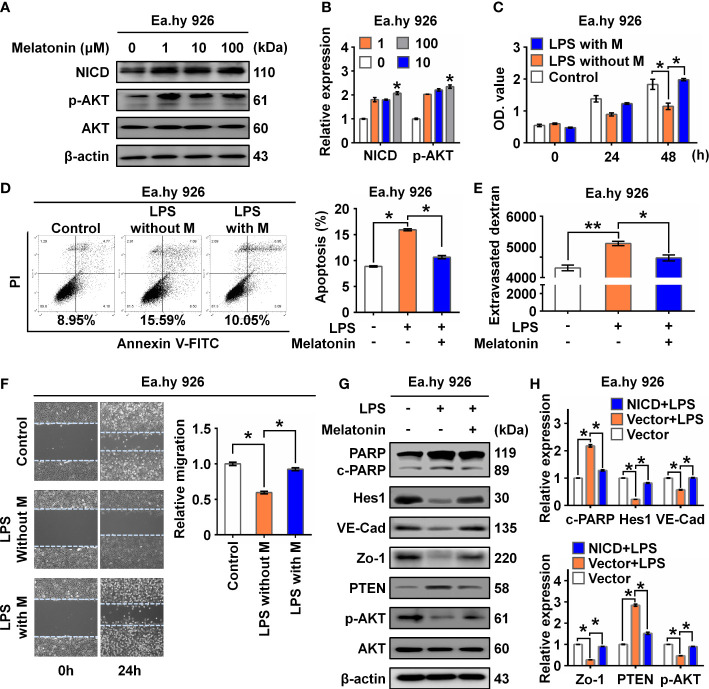
Melatonin alleviated LPS-induced endothelial dysfunction. **(A, B)** The level of NICD and p-AKT in Ea.hy 926 cells treated with indicated concentrations of melatonin were tested with Western blot. Representative images **(A)** and three experiments replicates **(B)** were displayed. **(C)** CCK8 assay was used to evaluate the proliferation of Ea.hy 926 cells treated with the control, LPS without melatonin or LPS with melatonin for indicated time. **(D)** The apoptosis of Ea.hy 926 cells treated with indicated conditions tested with Annexin V-FITC/PI staining by flow cytometry. Representative images **(D)**-left) and statistical analysis of apoptosis **(D)**-right) for Ea.hy 926 cells treated with the control, LPS without melatonin or LPS with melatonin were shown. **(E)** The endothelial permeability was determined by the transwell assay after being treated with the control, LPS without melatonin or LPS with melatonin. **(F)** The migration of Ea.hy 926 cells was tested by wounding healing assay after being treated with the control, LPS without melatonin or LPS with melatonin. Representative images **(F)**-left) and statistical analysis of migration **(F)**-right) of Ea.hy 926 regulated by indicated treatment were shown. **(G, H)** The protein levels of cleaved PARP, Hes1, VE-Cad, Zo-1, PTEN, and p-AKT were tested in LPS-treated Ea.hy 926 cells with or without melatonin. Representative images **(G)** and three experiments replicates **(H)** were displayed. The data were obtained from three independent experiments. Data are shown as the mean ± SEM. *p < 0.05. **p < 0.01.

It was reported that melatonin could improve the soluble Aβ1-42-induced impairment of spatial learning and memory *via* the Notch1/Hes1 signaling pathway (24). Therefore, we assayed expression of NICD in Ea.hy 926 cells treated with increasing concentrations of melatonin (0, 1, 10, 100 µM). We found that melatonin upregulated the expression of NICD and activated AKT signaling in the Ea.hy 926 cells (**Figures 4A, B**). To investigate the effect of melatonin on EC function, we first treated cells with LPS for 24 h and then with melatonin for 4 h. We found melatonin ameliorated the alterations in both cell proliferation (**Figure 4C**) and cell apoptosis (**Figure 4D**) of Ea.hy 926 cells stimulated with LPS. We also observed that melatonin significantly decreased intercellular permeability and promoted cell migration induced by LPS (**Figures 4E, F**). Moreover, melatonin reversed the effects of LPS on cleaved PARP, as well as the changes in Hes1, VE-Cad, Zo-1, PTEN, and p-AKT expression (**Figures 4G, H**). These results reveal that melatonin can alleviate LPS-induced endothelial dysfunction by increasing cellular levels of NICD and activating AKT signaling.

### 3.5 LPS decreases NICD stability through downregulating USPexpression

**Figure 5:**
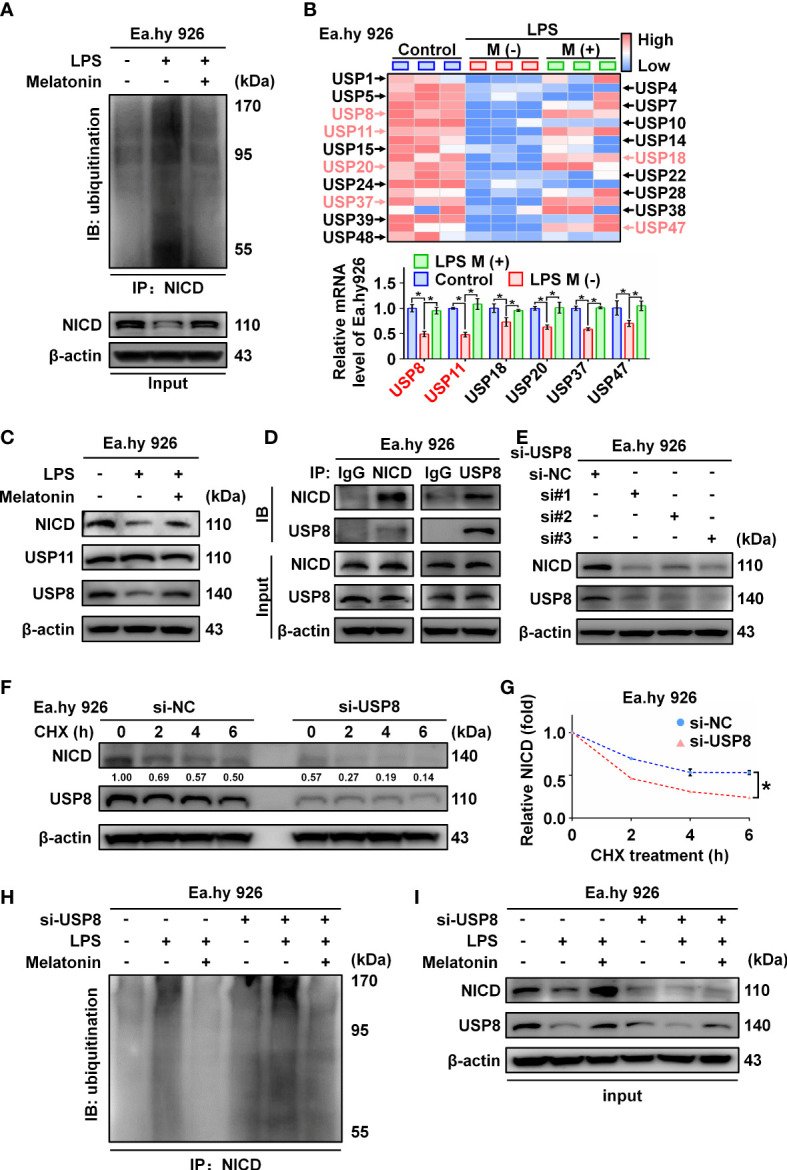
The ubiquitination of NICD was regulated by USP8. **(A)** Ubiquitinated NICD measured by immunoprecipitation with anti-NICD antibody and immunoblotting with anti-ubiquitin antibody in LPS treated Ea.hy 926 cells with or without melatonin. **(B)** The mRNA expression of a series of DUBs in Ea.hy 926 cells was tested by real-time qPCR. **(C)** The protein expression of USP8 and USP11 was detected by western blot in LPS-treated Ea.hy 926 cells with or without melatonin. **(D)** The interaction of NICD and USP8 in Ea.hy 926 cells was detected using Co-immunoprecipitation. **(E)** The expression of NICD and USP8 in Ea.hy 926 cells transfected with different USP8 siRNA. Scramble siRNA was used as negative control (si-NC). **(F, G)** The stability of NICD was tested in Ea.hy 926 cells transfected without or with si-USP8 at the indicated time after CHX (20 μM) treatment **(F)**. The quantitative analysis of **(G)** with Image J. **(H, I)** The ubiquitination of NICD in Ea.hy 926 cells with indicated treatment through NICD immunoprecipitation **(H)** and input **(I)**. Data are shown as the mean ± SEM. *p < 0.05. ns, no significance.

NICD is an intracellular domain protein generated by the cleavage activity of ADAM10 in the Notch pathway. We found that ADAM10 expression was not altered by LPS treatment (**Figure 1B**). Thus, we hypothesized that LPS likely promotes ubiquitination of NICD to affect its stability. As expected, we observed that LPS increased the ubiquitination of NICD, which was inhibited by melatonin (**Figure 5A**; **Supplementary Figure 2A**). Several studies have reported that NICD is highly ubiquitinated and regulated by deubiquitinating enzymes (DUBs) (25, 26). We investigated a series of deubiquitinating enzymes in Ea.hy 926 cells using RT-qPCR and observed that the mRNA levels of USP8 and USP11 decreased more than 2-fold in the LPS-treated cells compared with control cells (**Figure 5B**). Furthermore, Western blot data showed that USP8, but not USP11, was downregulated by LPS treatment (**Figure 5C**; **Supplementary Figure 2B**). We speculated that USP8 could interact with NICD to modulate NICD levels at the post-translational stage. The interaction between endogenous NICD and USP8 was validated by IP assays (**Figure 5D**, **Supplementary Figure 2C**).

Next, we knocked down USP8 expression in Ea.hy 926 cells using siRNA, which resulted in decreased expression of NICD (**Figure 5E**; **Supplementary Figure 2D**). To further confirm the function of USP8 in terms of NICD stabilization, Ea.hy 926 cells transfected with negative control siRNA or si-USP8 were treated with CHX for the indicated times (**Figure 5F**; **Supplementary Figure 2E**). Compared with the presence of USP8, the half-life of endogenous NICD was significantly shortened in USP8 knockdown cells (**Figure 5G**). To verify whether si-USP8 inhibited the stability of NICD by increasing NICD ubiquitination, we measured the ubiquitination of NICD in Ea.hy 926 cells with or without si-USP8 transfection. NICD exhibited more pronounced ubiquitination in the USP8 knockdown cells, and the LPS-induced ubiquitination of NICD was rescued by melatonin, regardless of USP8 expression (**Figures 5H, I**; **Supplementary Figures 2F, G**). In summary, these data demonstrate that LPS decreases the stability of NICD through downregulating the expression of USP8.

### 3.6 Melatonin attenuates sepsis-induced endothelial dysfunction *in vivo*

**Figure 6:**
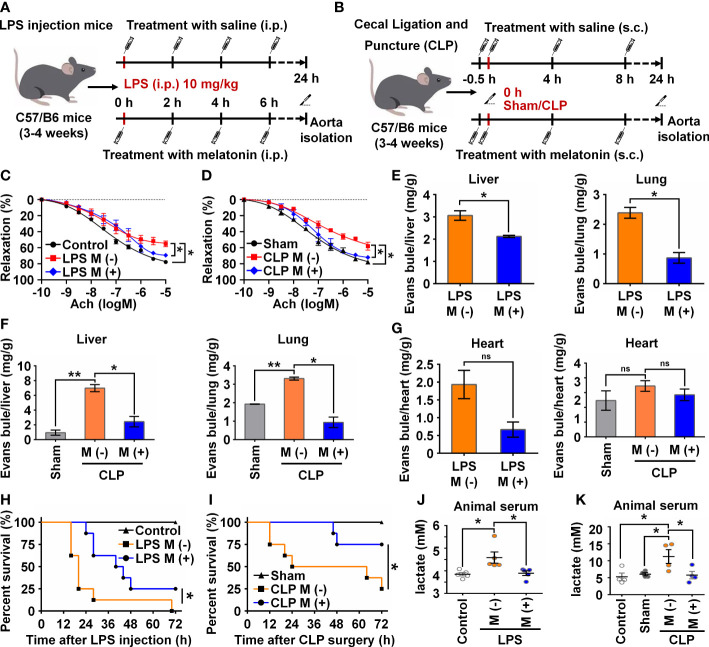
Melatonin attenuated sepsis-induced endothelial dysfunction. **(A, B)** Animal experiment flow chart in LPS group **(A)** and CLP group **(B)**. **(C, D)** The endothelium-dependent relaxation function of vessels was measured in LPS group treated without or with melatonin **(C)** and in CLP group treated without or with melatonin **(D)**. **(E)** Evans blue experiment in LPS group treated without or with melatonin was analyzed to test the vascular permeability function of liver (left) and lung (right). **(F)** Evans blue experiment in CLP group treated without or with melatonin was analyzed to test the vascular permeability function of liver (left) and lung (right). **(G)** Evans blue experiment in LPS group (left) and CLP group (right) treated without or with melatonin was analyzed to test the vascular permeability function of heart. **(H, I)** The survival rate of LPS group **(H)** and CLP group **(I)** treated with or without melatonin was analyzed (n=8 in each group). **(J, K)** The serum lactate level in LPS group **(J)** and CLP group **(K)** was tested by a lactic acid assay kit. Data are shown as the mean ± SEM. *p < 0.05. **p < 0.01. ns, no significance.

**Figure 7:**
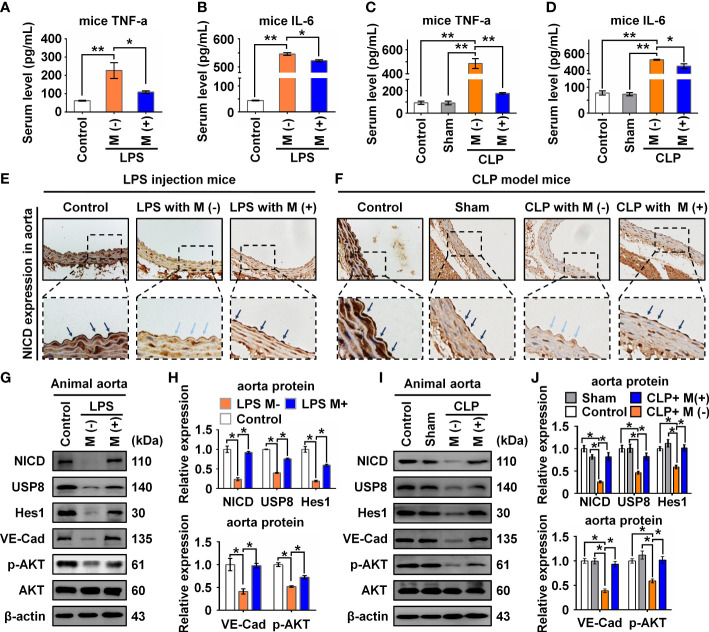
Melatonin attenuated sepsis-induced NICD downregulation in sepsis mice. **(A, B)** The serum levels of TNF-α **(A)** and IL-6 **(B)** in mice with the indicated treatment in LPS injection mice were detected by ELISA. **(C, D)** The serum levels of TNF-α **(C)** and IL-6 **(D)** in mice with the indicated treatment in CLP group were detected by ELISA. **(E, F)** Representative images of NICD expression in aorta tissues were detected by immunohistochemistry with indicated treatment in LPS group **(E)** and CLP group **(F)**. **(G, H)** The expression of NICD, USP8, Hes1, VE-Cad, and p-AKT in LPS group in aorta tissues were tested using Western blot analysis. Representative images **(G)** and three experiments replicates **(H)** were displayed. **(I, J)** The expression of NICD, USP8, Hes1, VE-Cad, and p-AKT in CLP group mice aorta tissues were tested using Western blot analysis. Representative images **(I)** and three experiments replicates **(J)** were displayed. Data are shown as the mean ± SEM. *p < 0.05. **p < 0.01.

To verify the functional effects of melatonin on vascular ECs in sepsis *in vivo*, we intraperitoneally injected LPS and used the CLP method to establish two sepsis models (27). In the LPS-induced sepsis model, the C57BL/6 mice were divided into three groups: Control, LPS with saline (M-), and LPS with melatonin (M+). Both saline and melatonin were injected every 2 h (**Figure 6A**), respectively. C57BL/6 mice in the CLP group were divided into four groups: Control, Sham, CLP with saline (M-), and CLP with melatonin (M+). Saline and melatonin were injected 0.5 h before CLP surgery, just after surgery, and 4 h and 8 h after surgery (**Figure 6B**). To examine changes in endothelium-dependent vasodilation in septic mice, the responses of arteries to acetylcholine (Ach) were measured. Compared with the control group, thoracic aorta vasodilation was significantly reduced in septic mice (**Figures 6C, D**), which were improved by melatonin treatment. To investigate whether melatonin contributes to vascular barrier function of septic mice, we examined EC permeability by measuring Evans Blue dye (EBD) penetration into tissues (liver, lung and heart). Our data showed that melatonin obviously reduced sepsis-induced vascular permeability as evidenced by the decreased penetration of EBD in liver and lung (**Figures 6E, F**), but not heart (**Figure 6G**). Of note, We observed that melatonin significantly rescued the death of septic mice, compared with vehicle-treated septic mice (**Figures 6H, I**). In addition, sepsis significantly increased serum levels of lactate, TNF-α and IL-6, which were effectively decreased by melatonin administration (**Figures 6J, K**, **7A–D**). The aortic mRNA levels of TNF-α and IL-6 were also obviously upregulated in LPS injection (**Supplementary Figures 3A, B**) and CLP group (**Supplementary Figures 3C, D**). Next, aortas were isolated to detect the level of NICD using immunohistochemistry and protein levels of NICD, USP8, Hes1, VE-Cad, and p-AKT in indicated groups by immunoblotting. As shown in **Figures 7E, F**, sepsis decreased NICD in the aortas, which were rescued by melatonin treatment. Immunoblotting results showed that melatonin recovered the expression of NICD, USP8, Hes1, VE-Cad, and p-AKT in aorta tissues of septic mice (**Figures 7G, H**). Similarly, sepsis-induced downregulation of NICD and its downstream molecules were rescued by melatonin (**Figures 7I, J**). These data suggest that melatonin could be used to attenuate sepsis-induced endothelial dysfunction and promote the survival rate.

## 4 Discussion

In this study, we found that LPS, IL-6, or septic serum could inhibit NICD and its downstream signaling molecules, resulting in impaired endothelial barrier function and EC apoptosis. Mechanistically, LPS decreased the stability of NICD through downregulating the expression of USP8. Overexpression of NICD or the application of the NICD-activating drug melatonin alleviated LPS-induced endothelial dysfunction. The therapeutic effects of melatonin on endothelial dysfunction in sepsis were mediated by upregulating USP8 expression and inhibiting NICD degradation, maintaining the stability of Notch signaling. Therefore, melatonin could be used to treat sepsis-mediated endothelial dysfunction by targeting NICD.

Endothelial dysfunction is a critical event in the pathophysiology of sepsis, which can lead to organ failure by enhancing vascular permeability, fomenting coagulation cascade activation and tissue edema, and compromising organ perfusion. Notch receptors seemingly play opposite roles in the endothelium and a majority of research has focused on the expression of Notch but not NICD, which is the functional downstream molecule of Notch activation. Numerous studies have shown that Notch activity in ECs is necessary to maintain endothelial barrier function and smooth muscle contractile phenotype, mainly through synergistic interactions with other pathways to coordinate vascular morphogenesis, differentiation, and function. *In vitro* experiments have shown that overexpression of the Notch ligand Jagged1 can promote EC proliferation (28), and activation of the Notch4 receptor can inhibit EC apoptosis (29). Notch signaling can also influence the kinetics of VE-Cad in retinal blood vessels (30).

To investigate the role of the Notch1 signaling pathway in sepsis-induced vascular injury, we used a Notch1 receptor-targeted drug, TGN, to inactivate the Notch1 signaling pathway. Previous studies have shown that TGN can inhibit the expression of Notch1, Hes1, and Hey1 in tumor cells but does not affect the expression levels of Notch2 and Notch3 (31). NICD and Hes1 expression were significantly decreased by TGN in this study. Furthermore, vascular EC injury caused by TGN was similar to that of LPS-induced vascular EC injury, indicating that downregulation of the Notch1 signaling pathway is involved in LPS-induced vascular EC injury. Patenaude et al. revealed that the inhibition of Notch signaling could lead to the loss of functional blood vessels, reducing endothelial carbon monoxide and nitrogen synthetase production and further inhibiting tumor growth and angiogenesis (32). Previous reports and our study consistently showed that inactivation of the Notch1 signaling pathway in vascular EC could lead to endothelial dysfunction, suggesting that LPS induces vascular endothelial injury through downregulating the Notch1 signaling pathway.

Yet the multi-function of Notch signaling in the same disease, even in the same cell is undeniable. For example, EC junctional integrity is impaired not only upon loss of Notch function but also due to sustained overactivation of endothelial Notch1. There are also conflicting data regarding the role of endothelial Notch and inflammation. On one hand, it has been reported that endothelial Notch activity reduces inflammation and is protective against atherosclerosis. In contrast, endothelial Notch signaling leads to higher expression of proinflammatory mediators, and inhibition of Notch reduces inflammation (33). In addition, Bai et al. found that the levels of Notch signaling molecules, including Notch1, Notch2, Hes1, and NICD, were increased in LPS-induced sepsis. Correcting the dysregulated Notch signaling pathway can rescue mice organ dysfunction and reduce inflammatory factor release (34). A likely explanation for the opposite expressions of NICD in macrophages and ECs during sepsis may be attributed to different Notch signaling downstream targets.

Here, we showed that the Notch1 signaling pathway was activated by overexpressing NICD in vascular ECs. Overexpression of Notch4 in ECs has also been shown to upregulate anti-apoptotic proteins to inhibit apoptosis induced by LPS. However, the effect of LPS on Notch4 expression in ECs was not mentioned. A similar study suggested that NICD could resist LPS-induced apoptosis of ECs. Further, while some studies detected the expression of the Notch receptor in LPS-treated vascular ECs, activation of the entire Notch signaling pathway was not reported. The innovation of this current study was to determine the activated form of Notch1 and its downstream effector molecules.

The Akt pathway has been associated with several human diseases and is mainly responsible for the response to extracellular signals, cell metabolism, proliferation, survival, and angiogenesis (23). In this study, we found that the Notch1 signaling pathway regulated the phosphorylation of Akt, which is mainly controlled by PI3K, PDK1, and PDK2. In addition, as a negative regulator of the Akt signaling pathway, PTEN can dephosphorylate PIP3, which is produced by PI3K, and Akt cannot be translocated to the cell membrane, making it accessible to phosphorylation (35). In this study, we found that the Notch1 signaling pathway negatively regulated PTEN expression and affected the phosphorylation level of Akt.

Melatonin is an endogenous compound in the human body that shares an amino acid sequence homologous to melatonin in cyanobacteria. Melatonin can also be used as a dietary supplemented (36). As a soluble substance, melatonin readily crosses cell membranes, binds to melatonin receptors in cells, and exerts various biological functions (37). Melatonin is metabolized rapidly, so melatonin is very safe at high doses with no significant clinical side effects. Studies have shown that melatonin can improve organ function and survival rate in various models of sepsis (38). Melatonin has been shown to improve hemodynamics in rats with endotoxemia by scavenging TNF-α in the plasma, nitric oxide synthase in the liver, and oxygen free radicals in the aorta (39). In a mouse model of sepsis, melatonin can alleviate blood-brain barrier dysfunction and brain edema through the SIRT-1 pathway (40). In the past, many studies have focused on melatonin’s anti-inflammatory and anti-oxidant effects on vascular ECs, but melatonin can also act as an inhibitor of the ubiquitin-proteasome system (41). We proposed that melatonin enhances the continuous expression of NICD and its downstream signaling molecules by inhibiting NICD ubiquitination. Consistent with our expectation, we observed that melatonin inhibited NICD ubiquitination and restored the decreased expression of USP8 induced by LPS, thus maintaining the stability of the Notch signaling pathway. USP8 has been suggested to exert protective effects against LPS-induced cognitive and motor deficits in mice by modulating microglial phenotypes *via* TLR4/MyD88/NF-κB signaling (42). However, the molecular mechanisms by which LPS regulates USP8 remain unknown. Considering that the mRNA levels of *Usp8* were downregulated in response to LPS, we hypothesized that post-transcriptional RNA modifications, such as m6A RNA methylation, could be involved in regulating *Usp8* levels. It has been demonstrated that methyltransferase-like 14-induced m6A modification participates in the regulation of USP48 in hepatocellular carcinoma by maintaining *Usp48* mRNA stability (43). Although emerging evidence has implicated a role for m6A modification in sepsis (44, 45), whether m6A modification directly associates with *Usp8* requires further exploration. In summary, our findings provide new insight into the molecular mechanisms by which melatonin improves sepsis-induced vascular injury, expanding our knowledge of melatonin in treating sepsis.

Since the Notch1 signaling pathway is essential to embryonic growth and development, complete or conditional knockout will cause high mortality in homozygous mice. As such, Notch1 knockout mice were not used in our study to verify the mechanism of Notch1 in vascular ECs *in vivo*. Notch1 knockdown has been limited to only *in vitro* experiments in previous studies, and Notch1 knockdown has not been reported in mice or animal models. In future studies, we plan to overexpress the NICD protein *via* an AAV approach that targets blood vessels to construct NICD conditional knockout mice to provide a more solid understanding of Notch1 *in vivo*.

**Figure 8:**
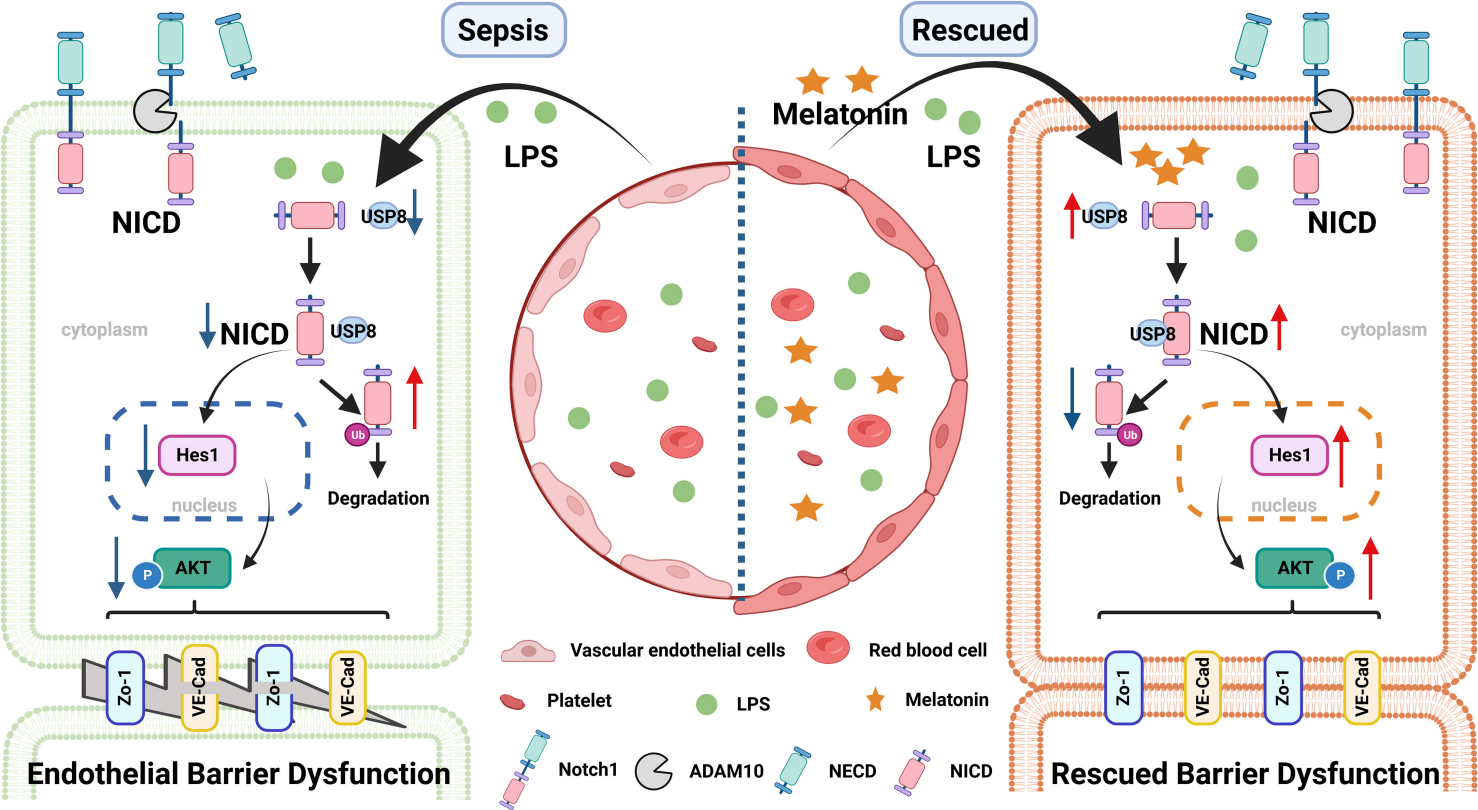
A pattern schematic created with BioRender. LPS promotes the ubiquitination of NICD by downregulating the level of USP8 to induce endothelial cell dysfunction in sepsis, which is rescued by melatonin.

In summary, our findings indicate that LPS-induced NICD downregulation and AKT inactivation may play important roles during endothelial dysfunction of sepsis. Our study also provides evidence that melatonin could be a potential therapeutic molecule in sepsis (**Figure 8**).

## Data availability statement

The raw data supporting the conclusions of this article will be made available by the authors, without undue reservation.

## Ethics statement

The studies involving human participants were reviewed and approved by the Research Ethics Board in the Children’s Hospital of Fudan University (IRB protocol number: 2020-424). Written informed consent to participate in this study was provided by the participants’ legal guardian/next of kin. The animal study was reviewed and approved by the Animal Studies Committee of the Children’s Hospital of Fudan University (IRB protocol number: 2019-321). Written informed consent was obtained from the owners for the participation of their animals in this study.

## Author contributions

TL, CZ, YW, and JY designed and carried out experiments and analyzed the data. TL, YZ, and GL wrote the manuscript. YZ and GL planned, designed, supervised, and coordinated the overall research efforts. All authors contributed to the article and approved the submitted version.
