# Supplementary material for: Inhibition of the intracellular domain of Notch1 results in vascular endothelial cell dysfunction in sepsis
Source: Front Immunol. 2023 May 2;14:1134556. doi: 10.3389/fimmu.2023.1134556 (PMC10185824; doi:10.3389/fimmu.2023.1134556)
Supplement: Supplementary file 1 [file DataSheet_1.docx]

**Supplementary Figure legends**

**
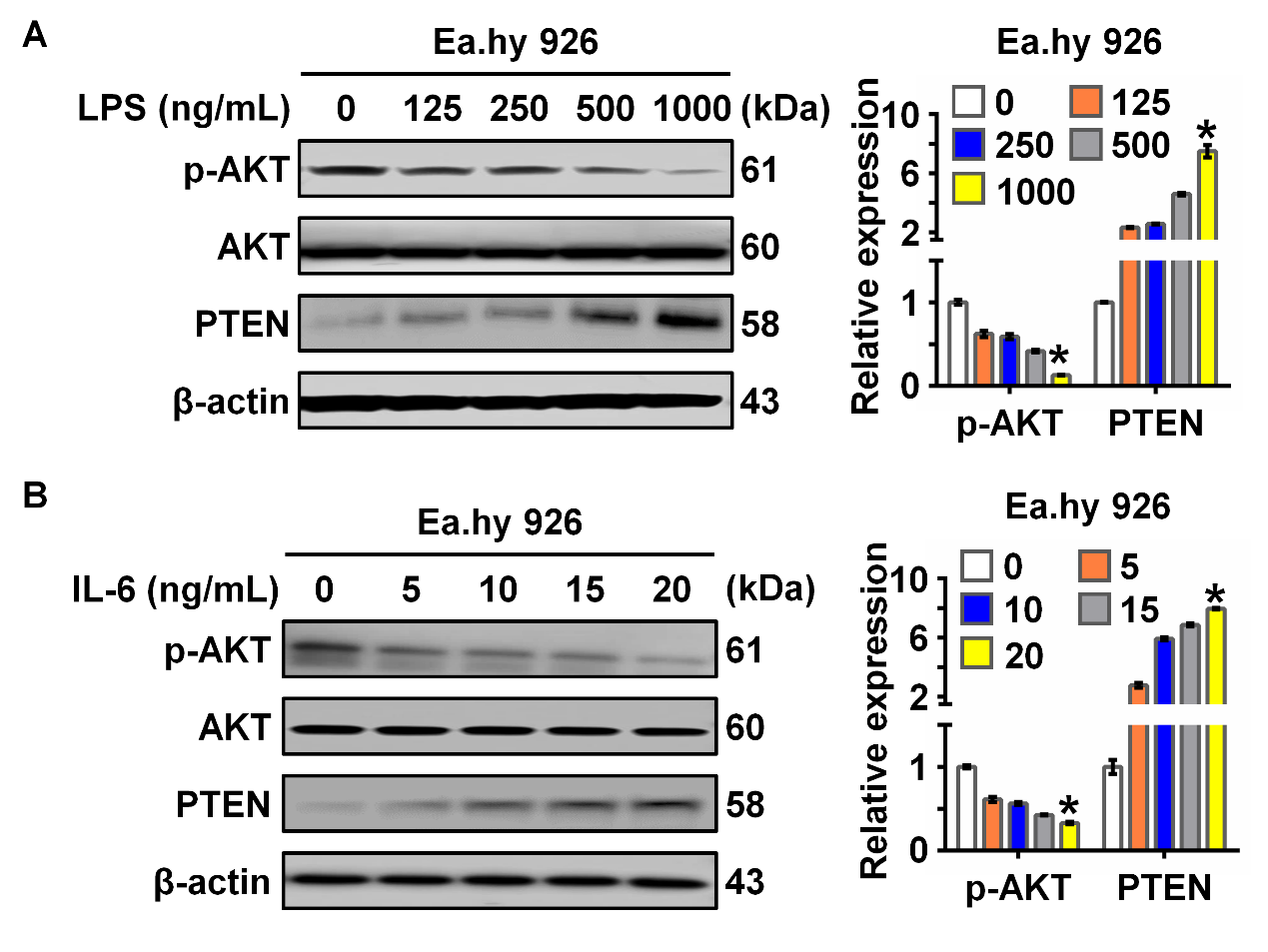
**

**Supplementary Figure 1** **LPS and IL-6 inhibited the activation of AKT pathway.** (A) The levels of p-AKT and PTEN were tested by Western blot in Ea.hy 926 cells treated with indicated concentration of LPS. Representative images (left) and three experiments replicates (right) were displayed. (B) The levels of p-AKT and PTEN were tested by Western blot in Ea.hy 926 cells treated with indicated concentration of IL-6. Representative images (left) and three experiments replicates (right) were displayed. Data are shown as the mean ± SEM. **p* < 0.05.

**
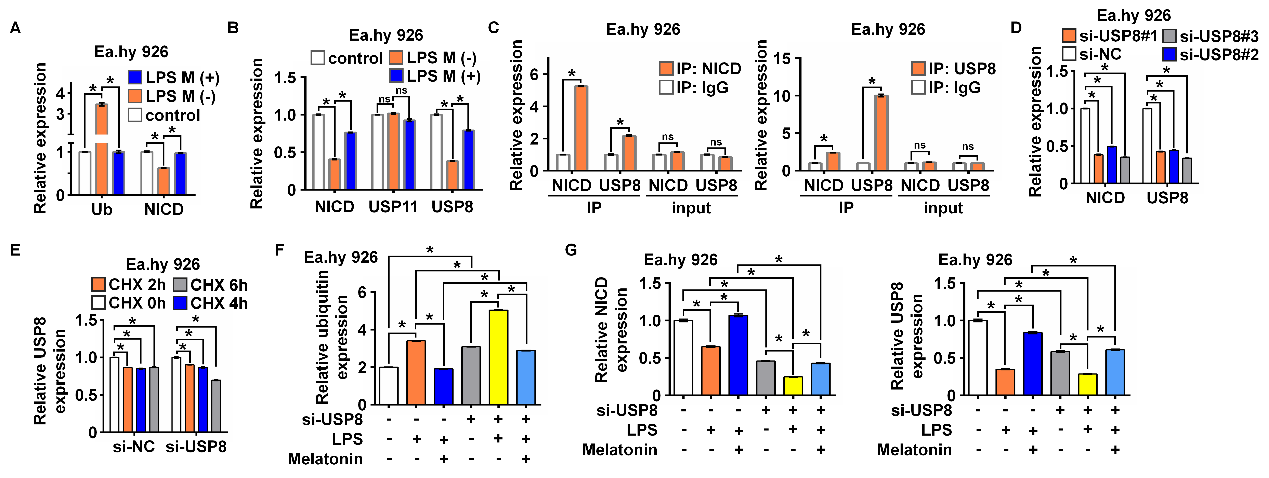
**

**Supplementary Figure 2 The quantitive analysis of NICD ubiquitination was regulated by USP8.** (A) The quantitive analysis of the NICD ubiquitination. (B) The quantitive analysis of the expression of .NICD, USP11 and USP8 in Ea.hy 926 cells with indicated treatment. (C) The quantitive analysis of the level of .NICD and USP8 in Ea.hy 926 cells by co-immunoprecipitation. (D) The quantitive analysis of the level of .NICD and USP8 in Ea.hy 926 cells transfected with siRNA. (E) The quantitive analysis of the level of USP8 in Ea.hy 926 cells transfected with siRNA with CHX treated. (F) The quantitive analysis of the level of ubiquitination in Ea.hy 926 cells transfected without or with si-USP8 by immunoprecipitation with NICD. (G and H) The quantitive analysis of the level of NICD (G) and USP8 (H) in Ea.hy 926 cells transfected without or with si-USP8. Data are shown as the mean ± SEM. **p* < 0.05. ns, no significance.

**
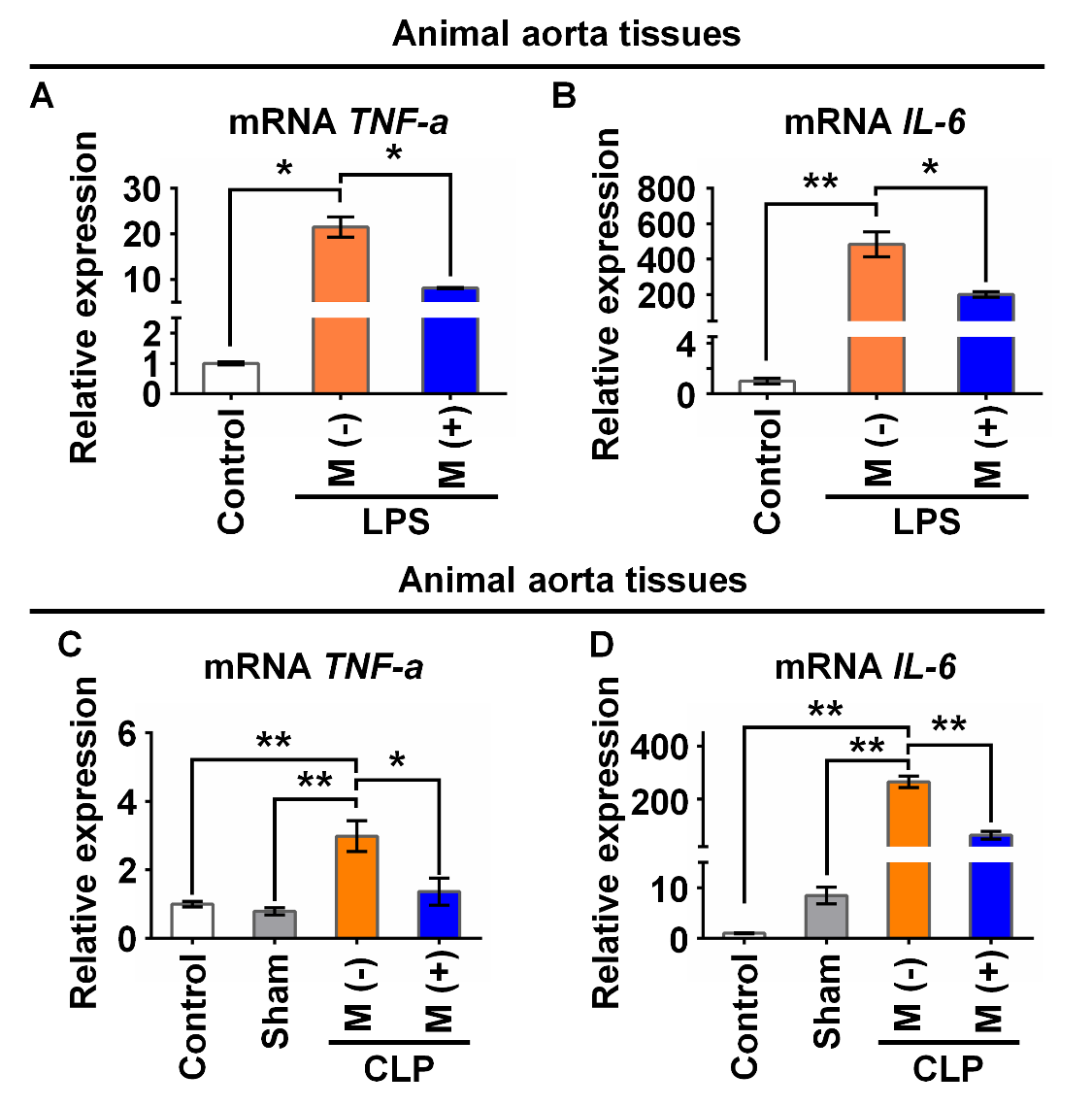
**

**Supplementary Figure 3 The mRNA levels of *TNF-α* and *IL-6* in aorta tissues of sepsis mice.** (A and B) The mRNA levels of *TNF-α* (A) and *IL-6* (B) in aorta tissues of LPS group with the indicated treatment were detected by RT-qPCR. (C and D) The mRNA levels of *TNF-α* (C) and *IL-6* (D) in aorta tissues of CLP group with the indicated treatment were detected by RT-qPCR. Data are shown as the mean ± SEM. **p* < 0.05. ***p* < 0.05.

**
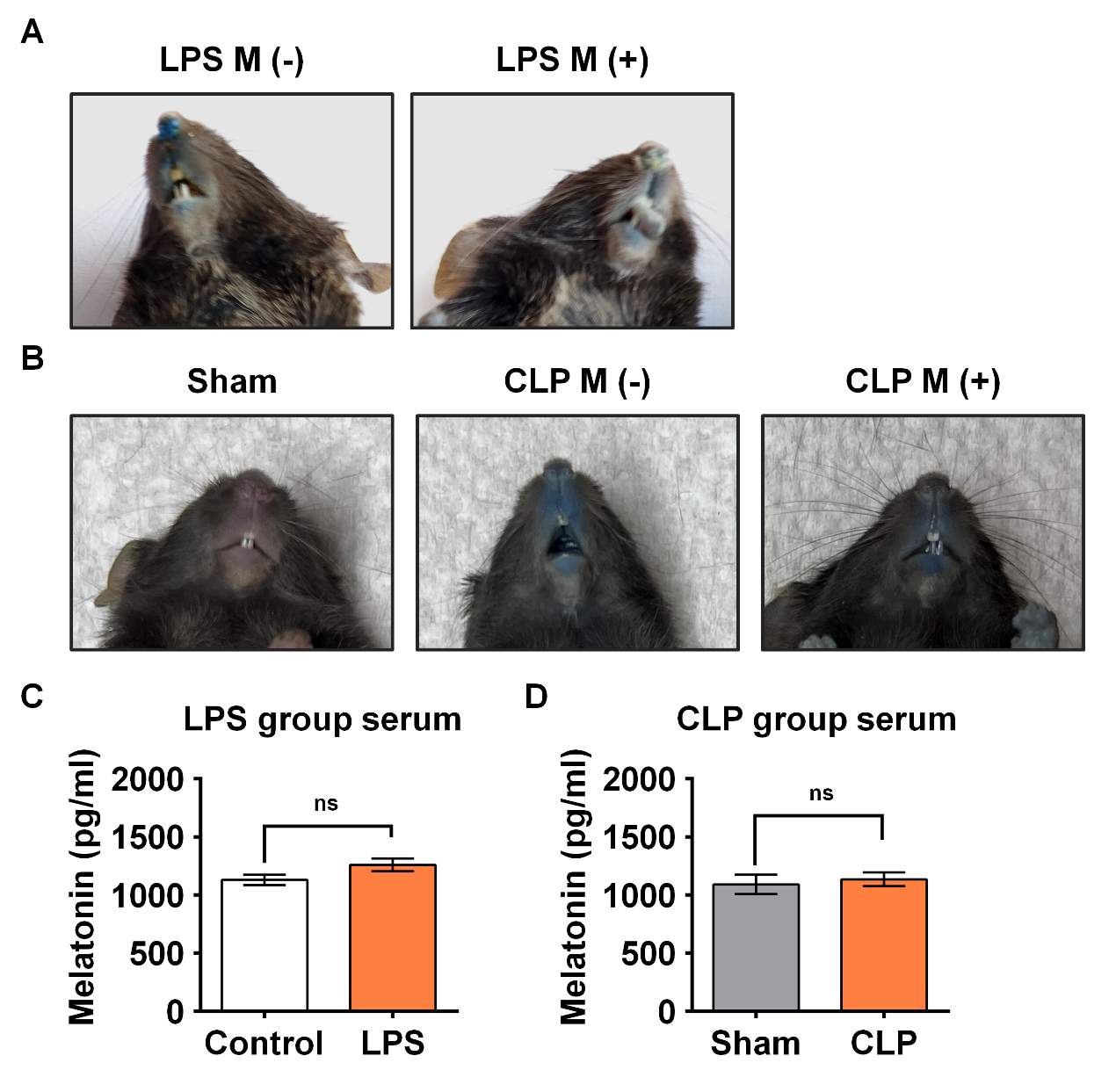
**

**Supplementary Figure 4 Evans blue dye and quantitive analysis of serum melatonin in mice.** (A and B) Representative images of evans blue dye in LPS group (A) or CLP group (B) without or with melatonin were displayed. (C and D) The quantification of serum melatonin in mice with indicated treatment. Data are shown as the mean ± SEM. ns, no significance.
