# Supplementary material for: Inhibition of the intracellular domain of Notch1 results in vascular endothelial cell dysfunction in sepsis
Source: Front Immunol. 2023 May 2;14:1134556. doi: 10.3389/fimmu.2023.1134556 (PMC10185824; doi:10.3389/fimmu.2023.1134556)
Supplement: Supplementary file 2 [file Table_1.docx]

**Supplemental Table 1. The sequences of primers for qRT-PCR (5′-3′)**

| **Primer name** | **Forward or Reverse primer** | **Sequence (5′-3′)** |
| --- | --- | --- |
| *Homo* USP1 | Forward | GCTGCTAGTGGTTTGGAGTTT |
|  | Reverse | GCATCACAACCGCAAATAATCC |
| *Homo* USP4 | Forward | TTTCCTGGCCCAATAGACAAC |
|  | Reverse | GGTAGGGACCAATACATAGTCCA |
| *Homo* USP5 | Forward | GCTGCTGTCAGTATTACCGAC |
|  | Reverse | AAAGCCCAGAAACGTGTTCATA |
| *Homo* USP7 | Forward | GATGAAAAGTCGTTCAGTCGTCG |
|  | Reverse | TTTGAATCCCACGCAACTCCA |
| *Homo* USP8 | Forward | GTCCAGGAGTCACTGCTAGTT |
|  | Reverse | AGGAGCCAGTTTTCATAGCCT |
| *Homo* USP10 | Forward | GAGGGCACAGCTACCAACG |
|  | Reverse | AGGGGAGATATGGCGGGAG |
| *Homo* USP11 | Forward | CGTTTCCGGGACCAGAATCC |
|  | Reverse | CATCGCCGTCCGTTCTCTTC |
| *Homo* USP14 | Forward | ATGCCGCTCTACTCCGTTACT |
|  | Reverse | GCCTTGAATACCATTGGAGGTTC |
| *Homo* USP15 | Forward | CGACGCTGCTCAAAACCTC |
|  | Reverse | TCCCATCTGGTATTTGTCCCAA |
| *Homo* USP18 | Forward | CCTGAGGCAAATCTGTCAGTC |
|  | Reverse | CGAACACCTGAATCAAGGAGTTA |
| *Homo* USP20 | Forward | GGTTGCCTGCCCCTATGTT |
|  | Reverse | GGTTCACGGTCAAGTTGTGC |
| *Homo* USP22 | Forward | CTGGGACATCAGCTTGGATCT |
|  | Reverse | CTTTCCCCGTTTACCACGTTG |
| *Homo* USP24 | Forward | TGGACGCGGAGAAGAATGATG |
|  | Reverse | CTCGCTTGTAAGGGATGGACC |
| *Homo* USP28 | Forward | CACTGTTGCTACAGAACCATCT |
|  | Reverse | TGGGAGACTCCAGTAGACTCA |
| *Homo* USP37 | Forward | TTCTTGGTAATCCGGGTAGAGG |
|  | Reverse | CAGTACGATTTTCTAGCAACCCT |
| *Homo* USP38 | Forward | CAGTGCGAGGCCATGTTTG |
|  | Reverse | CGGTGGTATCGTGCGTAGG |
| *Homo* USP39 | Forward | CACTTACCTGCCGGGTATTGT |
|  | Reverse | CCTGGAGGACGTTTGATGTTCT |
| *Homo* USP47 | Forward | AACCACAGATGTTACAAGGAGC |
|  | Reverse | AAGATATGTGTCGATTCGCCAG |
| *Homo* USP48 | Forward | CTTCGGCAGGCACTCTACTTA |
|  | Reverse | ATGCTCACAAATTGTTTGAGGC |
| *Homo* ACTB | Forward | CATGTACGTTGCTATCCAGGC |
|  | Reverse | CTCCTTAATGTCACGCACGAT |
| *Mus* TNF-α | Forward | CTGCAAGAGACTTCCATCCAG |
|  | Reverse | AGTGGTATAGACAGGTCTGTTGG |
| *Mus* IL-6 | Forward | CAGGCGGTGCCTATGTCTC |
|  | Reverse | CGATCACCCCGAAGTTCAGTAG |
| *Mus* GAPDH | Forward | AGGTCGGTGTGAACGGATTTG |
|  | Reverse | GGGGTCGTTGATGGCAACA |
